# Combined monitoring of IgG and IgA anti-Spike and anti-Receptor binding domain long term responses following BNT162b2 mRNA vaccination in Greek healthcare workers

**DOI:** 10.1371/journal.pone.0277827

**Published:** 2022-11-21

**Authors:** Ioannis Sarrigeorgiou, Dimitra Moschandreou, Alexios Dimitriadis, Gerasimina Tsinti, Evangelia Sotiropoulou, Eleni Ntoukaki, Petros Eliadis, Marija Backovic, Stavroula Labropoulou, Nicolas Escriou, Abraham Pouliakis, Georgia Giannopoulou, Eleni Gaitanarou, Konstantinos Lazaridis, Andreas Mentis, Avgi Mamalaki, Elisavet Grouzi, Peggy Lymberi

**Affiliations:** 1 Immunology Laboratory, Immunology Department, Hellenic Pasteur Institute (HPI), Athens, Greece; 2 Department of Transfusion Service and Clinical Hemostasis, "Saint Savvas" Oncology Hospital, Athens, Greece; 3 Biotechnology Unit, HPI, Athens, Greece; 4 Laboratory of Molecular Biology and Immunobiotechnology, HPI, Athens, Greece; 5 Structural Virology Unit, Department of Virology, Institut Pasteur, Paris, 75015, France; 6 Diagnostic Services Department, HPI, Athens, Greece; 7 Innovation Lab, Vaccines, Department of Virology, Institut Pasteur, Paris, 75015, France; 8 Second Department of Pathology, National and Kapodistrian University of Athens, "ATTIKON" University Hospital, Athens, Greece; Central University of Tamil Nadu, INDIA

## Abstract

Studies on the humoral response to homologous BNT162b2 mRNA-vaccination focus mainly on IgG antibody dynamics, while long-term IgA kinetics are understudied. Herein, kinetics of IgG and IgA levels against trimeric-Spike (S) and Receptor-Binding-Domain (RBD) were evaluated by in-house ELISAs in 146 two-dose vaccinated Greek healthcare workers (HCWs) in a 9-month period at six time points (up to 270 days after the first dose). The effect of a homologous booster third dose was also studied and evaluated. The peak of immune response was observed 21 days after the second dose; 100% seroconversion rate for anti-S and anti-RBD IgG, and 99.7% and 96.3% respectively for IgA. IgG antibody levels displayed higher increase compared to IgA. Declining but persistent anti-SARS-CoV-2 antibody levels were detected 9 months after vaccination; IgG and IgA anti-S levels approached those after the first dose, while a more rapid reduction rate for anti-RBD antibodies led to significantly lower levels for both classes, supporting the need for a booster dose. Indeed, a homologous booster third dose resulted in enhanced levels of anti-S of both classes, whereas anti-RBD didn’t exceed the peak levels after the second dose. Previous SARS-CoV-2 infection, flu vaccination, BMI<35 and the occurrence of an adverse event upon vaccination, were associated with higher IgG antibody levels over time, which however were negatively affected by age increase and the presence of chronic diseases. Overall, after concurrently using the S and RBD target-antigens in in-house ELISAs, we report in addition to IgG, long-term persistence of IgA antibodies. Regarding antibody levels, homologous mRNA vaccination gives rise to an effective anti-viral protection up to 9 months negatively correlated to age. Considering that COVID-19 is still a matter of public concern, booster vaccine doses remain critical to vulnerable individuals.

## Introduction

Pfizer-BioNTech (BNT162b2) vaccine was one of the two mRNA-based vaccines (along with the mRNA-1273 vaccine by Moderna) against COVID-19, which received Emergency Use Authorization by the U.S. Food and Drug Administration (FDA), in December 2020. The mRNA carried by those vaccines encodes the full-length viral spike (S) ectodomain of SARS-CoV-2 [1]. It is considered critical that BNT162b2 vaccine provokes a strong immune response against the S protein, and particularly its Receptor Binding Domain (RBD), which preferentially binds the angiotensin-converting enzyme 2 (ACE2) receptors for viral entry in host cells [2]. Antibody (Ab) measurement can usually validate an efficient immune response upon vaccination, as well as the duration of anti-viral protection [3]. Commercial or in-house serological assays measuring serum Abs against the S protein or its fragments S1 and S1/RBD are currently globally used to evaluate the effectiveness of the vaccination [4,5]. These fragments contain target epitopes for neutralizing Abs [6,7]. Despite high vaccine coverage and effectiveness, the incidence of symptomatic infection with SARS-CoV-2 has been increasing in Greece, in Europe as well as worldwide. Whether the increasing incidence of infection is due to waning immunity after vaccination or emerging virus mutants is still unclear as different factors such as homologous vs heterologous vaccination, vaccine type, age, may affect the outcome [8,9].

Studies on humoral response to mRNA vaccines mostly use commercial assays, employing different capture target-antigens, and focusing on specific IgG Ab dynamics [4,5,10,11] whereas, fewer studies are addressing the long-term dynamics of IgA response [12–16]. For example, some studies have used commercially available kits measuring anti-trimeric Spike or anti-RBD IgG in correlation with anti-S1 IgA [13], while others used kits comparing anti-RBD IgG kinetics with the levels of IgA Abs targeting a mixture of S1/S2 with Nucleocapsid (N) protein [14,17,18].

In this study we explored the IgG and IgA Ab responses over 9 months after homologous BNT162b2 vaccination in a cohort of 146 Health Care Workers (HCWs) of a Greek hospital, using in-house ELISAs, providing the opportunity for thorough analyses. The effect of a homologous booster third dose was also evaluated, extending our serological analysis up to 10 months. The combined measurement of Abs against intact S protein (trimeric S) and its RBD fragment allowed exploring the diversity of responses among the vaccinated HCWs, with respect to their distinct demographic characteristics and clinical profiles. Moreover, the additional use of in-house ELISAs to identify IgG and/or IgA Abs to N antigen indicated the potential asymptomatic (or COVID-19) individuals among the studied HCWs.

## Materials & methods

### Clinical data

One hundred and forty-six (146) participants (male/female: 41/105, age range: 21–65 median: 49) were enrolled in a prospective study evaluating the kinetics of anti-SARS-CoV-2 Abs after vaccination with the Pfizer-BioNTech (BNT162b2) SARS-CoV-2 vaccine. All the subjects were HCWs of the “Saint Savvas” Oncology Hospital in Athens, Greece. HCWs have been prioritized for vaccination since January 2021 and, therefore, mature data for the Ab responses at 9 months following vaccination have been collected. Demographic data of our HCWs cohort are presented in **Table 1**. Individuals were divided into three age groups: younger than 40 years old; 40–50 years old; and 50–65 years old. Body mass index (BMI) was calculated using each individual’s weight and height data. Based on BMI, the subjects were divided into two groups: Individuals with a BMI of less than 35 and overweight individuals with a BMI of 35 or more. Previous annual flu vaccination and pneumococcal vaccination in the last 1 year were also considered. The medical history of the subjects included various chronic diseases, as presented in **Table 2,** classified into the following groups: cardiovascular disease (e.g., hypertension, coronary artery disease), diabetes, hypercholesterolemia, bronchial asthma, autoimmune diseases (e.g., Hashimoto’s thyroiditis, rheumatoid arthritis), benign tumors (e.g., pituitary adenoma), malignancies (e.g., breast cancer), hematologic diseases and others (e.g., migraines). None of the female participants was pregnant or lactating. Post-vaccination adverse events are presented in **Table 3**, classified into the following groups: local effects (e.g., pain or swelling at the vaccination site, limitation of hand movement), fatigue, arthralgias/ myalgias/ chills/ fever, headache, digestive system disorders (e.g., vomiting, diarrhea) and others (e.g., malaise). The study was in accordance with the Declaration of Helsinki and International Conference for harmonization for good clinical practice and was approved by the Scientific and Ethics Committee of “Saint Savvas” Oncology Hospital of Athens (Ref No. 186/448-140-701/07-01-2021). All subjects gave written informed consent, which was signed before participating in the study. Subject data were kept confidential in accordance with the rules of the General Data Protection Regulation.

**Table 1:** Characteristics of individuals included in the study.

Characteristics	Number of subjects (n), percentage (%)
**Number of individuals**	**146**
Age: median, SD, range interval	49y, ±12y, 21–65y
Age ≤ 40 (n, %)	38 (26%)
Age [40–50] (n, %)	43 (29.5%)
Age [50–65] (n, %)	65 (44.5%)
Women (n, %)	105 (71.9%)
Men (n, %)	41 (28%)
BMI > 35 (n, %)	10 (6.8%)
Chronic diseases (n, %)	41 (28.1%)
SARS-CoV-2 PCR+ (n, %)	12 (8.2%)
Annual Flu vaccination	70 (47.9%)
Pneumococcal vaccination (within the last 1 year)	15 (10.2%)

**Table 2:** Chronic or other diseases of individuals included in the study.

Chronic Diseases	n = 39 (34.7%)
Cardiovascular disease	19 (13%)
Diabetes Mellitus type II	2 (1.4%)
Hypercholesterolemia	4 (2.8%)
Asthma	9 (6.2%)
Autoimmune Diseases	8 (5.4%)
Benign tumor	3 (2.1%)
Malignancies	2 (1.4%)
Hematologic diseases	2 (1.4%)
Other	2 (1.4%)

**Table 3:** Adverse events upon BNT162b2 vaccination.

Adverse Events	n = 95 (72.5%)
Local effects	80 (84%)
Fatigue	60 (63%)
Arthralgias/ myalgias/ chills/ fever	46 (48%)
Headache	48 (50%)
Digestive system disorders	7 (7%)
Other	10 (9.5%)

### Sample collection and data organization

For the development of the in-house ELISAs, serum samples from 40 COVID-19 patients were collected between January and April 2020, 1–2 months after nasopharyngeal swab collection and being tested positive with RT-PCR at the Hellenic Pasteur Institute, Diagnostic Services Department. Moreover, serum samples from 40 healthy donors of pre-COVID-19 era collected in 2017 were provided by the Blood Transfusion Department, University Hospital of Thessaly.

For the longitudinal study of vaccination effectiveness, blood samples from 146 HCWs were collected by vein puncture and serum was separated within 4h of blood collection and stored at -80°C until the day of measurement. All individuals received at least two vaccine doses, the second 21 days after the first. Blood samples were collected at six (6) time points, the first immediately before the first vaccination (**Day 0)** and then on **Day21** (day of second dose), **Day42, Day90, Day180** and **Day270**. Finally, one more sample (**Day21γ**) was collected from HCWs who had received a third dose of the vaccine, 21 days after the boost. A unique code was attributed to each participant; all serum samples were labeled and archived using a coding system.

### Antigens

The pcDNA3.1 plasmids encoding the SARS-CoV-2 S ectodomain (amino acids 1 to 1208) followed by a fold-on trimerization motif and tags (8×HisTag, StrepTag, and AviTag) and the RBD (amino acids 331 to 519), were kindly provided by Prof. Felix Rey (Structural Virology Research Unit, Institut Pasteur Paris) [5]. The pETM11 expression vector encoding the His-tagged SARS-CoV-2 N protein was kindly provided by Dr. Nicolas Escriou (Laboratoire d’Innovation: Vaccins, Institut Pasteur Paris) [5,18,19].

The trimeric S glycoprotein and its RBD fragment were produced by transient expression of exponentially growing Expi293 suspension cells (Thermo Fisher Scientific, Waltham, MA) using FectoPRO® transfection reagent (Polyplus) following the manufacturer’s guidelines. Recombinant proteins were purified by affinity chromatography using Strep-Tactin®XT 4flow column (IBA lifesciences) according to the manufacturer’s instructions. The His-tagged SARS-CoV-2 N protein was bacterially expressed in E. coli BL21 (DE3) and purified as a soluble dimeric protein by affinity purification using a Ni-NTA Protino column (Macherey Nagel). The eluates were analysed in 4–20% gradient precast protein gels (Nippon Genetics).

### Development of in-house ELISAs

Samples from the same donor at different time points of vaccination were measured in parallel for anti-S, anti-RBD and anti-N IgG and IgA Abs, using in-house ELISAs developed based on previously described methods [5]. Briefly, 30μl/well of N, S and RBD antigens were coated in high binding half-area ELISA microplates (675061-Greiner Bio-One, Kremsmünster, Austria) diluted at optimum concentrations (1, 2 and 3μg/ml, respectively), in carbonate-bicarbonate buffer 0.1M pH 9.6 and incubated overnight at 4°C. Plates were washed 4x with Phosphate-buffered saline (PBS) and saturated with 100μl/well PBS containing 5% w/v milk powder for 1h at 37°C. For IgG ELISAs, sera were diluted 1/100 for N and 1/400 for S and RBD, while for IgA ELISAs, sera were diluted 1/100 for all antigens. In each assay, the sera were diluted in sample dilution buffer (blocking buffer containing 0.05% Tween), 30μl/well were added and incubated for 2 h at 37°C. After extensive washing, alkaline phosphatase conjugated secondary Abs against human IgG (109-055-008, Jackson ImmunoResearch, Pennsylvania, USA) or IgA (109-055-011, Jackson ImmunoResearch) were added at 0.04 and 0.1μg/ml final concentration respectively (50μl/well) and incubated for 2 h at 37°C. After extensive washing, antibody binding was assessed with the substrate 4-nitrophenyl-phosphate-disodium salt hexahydrate (pNPP-N2765, Sigma Chemicals, St. Louis, MO, USA) and optical density (O.D.) was measured at 450nm (620nm reference measurement) with a TECAN photometer (TECAN Spark Control Magellan V2.2, Grödig/Salzburg, Austria). For inter assay normalization, three selected positive and three negative (unvaccinated and uninfected healthy donors) controls were used in every plate. Healthy donors of pre-COVID-19 era (n = 40) and COVID-19 patients (n = 40) were analyzed to determine optimal incubation time in comparison with negative control values and for the estimation of assays sensitivity and specificity. All the assays were compared in parallel with the FDA approved SARS-CoV-2 IgG & IgA ELISA KIT (EUROIMMUN Medizinische Labordiagnostika AG, Lübeck, Germany), according to the manufacturer’s instructions. The cut-off value for each in-house assay was determined using the mean O.D. plus 2.5 standard deviations of pre-pandemic healthy controls. The enzyme reaction was stopped when the mean value of three selected positive controls reached O.D. equal to 1.00 (~1h). These optimized ELISAs were used for sera screening. O.D. values were converted to Arbitrary Units (AU) per well (AU/well = ODx1000).

### Statistical analysis

All measured RAW data are included in **S1 Table.** The d’Agostino- Pearson omnibus normality test was used to assess the normality of the data distribution. For two independent group comparisons, the Mann–Whitney U test was used. The non-parametric Friedman test was used to detect differences in Ab levels across multiple time points, as well as analysis of the Ab rate of change (RC). Chi-square analysis was used for comparisons of nominal characteristics. To assess the effect of subject characteristics (i.e. sex, age, BMI, chronic diseases, adverse events after vaccination and simultaneous vaccination against flu or pneumococcus) on Ab levels, we applied multivariate linear regression models with the previous characteristics as multiple inputs and Ab levels for each time point (excluding day 0) as output. In order to investigate the Abs RC from Day42 up to Day270, we applied simple linear regression and calculated for each subject the line of best fit to Ab levels vs. time. The slope of these lines represents the drop rate and is expressed in antibody measurement units/day. Furthermore, we investigated if the Ab RC index is related to: sex, age, BMI, chronic diseases, adverse events after vaccination and simultaneous vaccination against flu or pneumococcus using multivariate linear regression. In all cases, the significance level was set at 5%, the tests were two sided and a result was considered significant if the estimated P-value (p) was less than the significance level. Statistical analysis was performed and graphs were made in GraphPad Prism version 6.0.0, (GraphPad Software, San Diego, California USA), as well as, the SAS for Windows 9.4 software platform (SAS Institute Inc., NC, U.S.A.).

## Results

### Validation of IgG and IgA ELISAs against SARS-CoV-2 Nucleocapsid, Spike and RBD antigens

**Fig 1:**
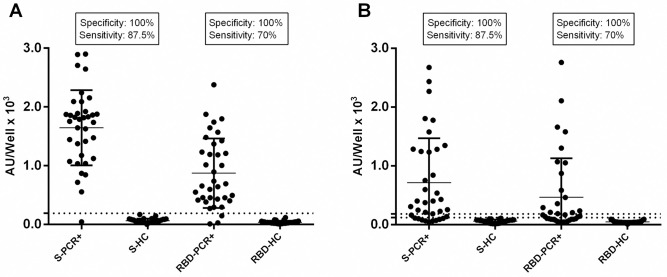
In-house ELISA validation. The sensitivity and specificity of the in-house ELISAs for IgG (A) and for IgA (B) Abs against S and RBD were estimated with 40 pre-pandemic sera (Healthy C.) and 40 PCR+ COVID-19 sera. For IgG anti-S and anti-RBD Abs the cut-off point was 150 AU/well (dashed line). For IgA Abs the cut-off point for anti-S was 120 AU/well and for anti-RBD 80 AU/well (dashed lines).

Pre-pandemic sera (n = 40) and sera from confirmed PCR+ COVID-19 recovered patients (n = 40) were analyzed for the validation of our in-house ELISAs. The same sera were tested in parallel with the EUROIMMUN IgG and IgA ELISA kit. All pre-pandemic sera exhibited low or no Ab binding against SARS-CoV-2 proteins in our in-house ELISAs and were negative at the EUROIMMUN test. All COVID-19 samples had high levels of both IgG and IgA anti-N Abs, indicative of the strong immunogenicity of N protein upon natural infection. Interestingly, some pre-pandemic sera exhibited low IgG binding capacity against the N protein probably due to cross-reactions arising from previous infections with other corona viruses leading to 400 AU/well cut off point for IgG and 170 AU/well for IgA Abs (**S1 Fig**). For the S and RBD target antigens, the in-house ELISA for IgG Abs displayed ≥92.5% sensitivity and ≥98% specificity, while the respective characteristics for IgA Abs ELISA were ≥70% and ≥99%, respectively **(Fig 1)**.

### Identification of asymptomatic pre-infected individuals

**Fig 2:**
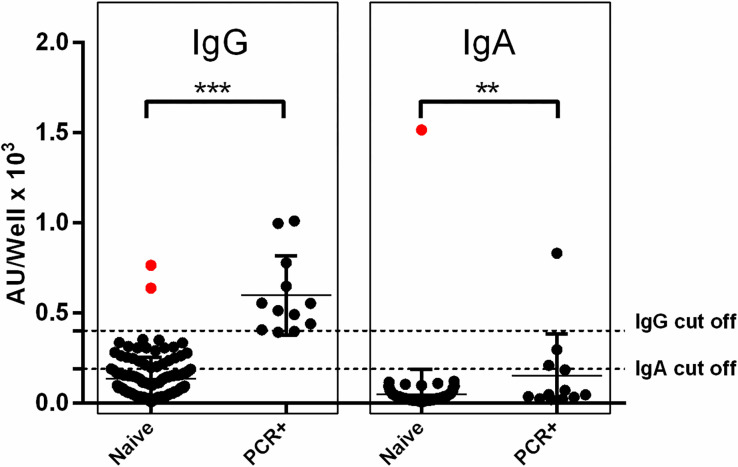
Serum anti-N IgG and IgA antibody levels prior to vaccination. For PCR- Naïve individuals (n = 134) and PCR+ (n = 12) individuals the sample prior to vaccination was on Day0. The cut-off point of IgG and IgA ELISAs was established at 400 AU/well and 170 AU/well, respectively (dashed lines). Asterisks (*) indicate statistically significant differences (*p*-value: **<0.01, ***<0.001) between the compared groups. Each dot represents an individual and error bars represent the mean value and standard deviation of the distribution.

To assess the serological responses of the 146 vaccinated donors against S and RBD, with or without history of documented COVID-19 infection, it was necessary to examine first pre-vaccination sera (Day0) in order to identify those who were asymptomatic, along with those with a known history of confirmed SARS-CoV-2 infection. To this end, we used the in-house anti-N ELISA for the detection of IgG and IgA Abs binding to SARS-CoV-2 N protein on Day0 (**Fig 2**). Among our HCWs cohort, the 12 PCR+ individuals exhibited high levels of IgG and/ or IgA anti-N Abs on Day0. Additionally, three individuals without any previous COVID-19 symptoms, were found, similarly to PCR+ group, to display Ab levels of IgG (n = 2) or IgA (n = 1) anti-N Abs above threshold. These three subjects along with the 12 PCR+ individuals were excluded from the analysis, and eventually 131 out of the 146 enrolled donors constituted the naïve group that evaluated with respect to anti-S and anti-RBD Ab responses.

### Anti-viral IgG kinetics in response to two-dose BNT162b2 vaccination

**Fig 3:**
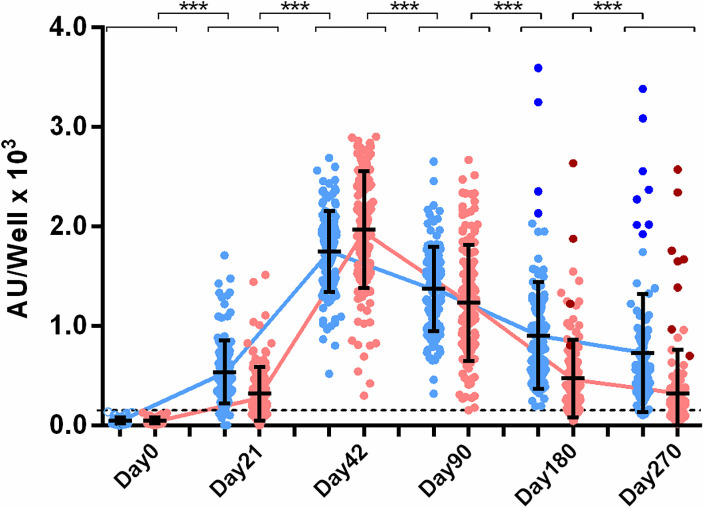
IgG anti-S and anti-RBD levels post-vaccination. Anti-S (blue) and anti-RBD (red) Abs were measured on Day 0, 21, 42, 90, 180 and 270 days after the first dose on Day0. Cut-off values for anti-S and anti-RBD Abs were 150 AU/well (dashed line). Dark colored dots represent post-vaccination infected individual’s anti-S and anti-RBD IgG levels respectively. Asterisks (***) indicate statistically significant differences (*p*-value<0.001) between the compared groups. Each dot represents an individual, and error bars represent the mean value and standard deviation of the distribution.

The anti-S IgG kinetics within a 9-month period are shown in **Fig 3**. On Day0 anti-S IgG mean levels were 50±31, on Day21 539±305 and on Day42 1730±392 AU/well marking a significant increase in Ab levels (p<0.001). The first vaccine dose had 92.3% (121/131 naïve HCWs) anti-S seropositivity (p<0.001), while the second dose resulted in 100% seroconversion. On Day90 anti-S IgG mean levels was 1362±419 AU/well, showing a decline of 21.9% from Day42 (p<0.001). On Day180, the levels dropped to 831±388 and on Day270 to 591±320 AU/well, a reduction of 56% and 70% from Day42, respectively (p<0.001). Interestingly 4/131 (3%) HCWs, in contrast to the rest of the group, exhibited higher anti-S levels on Day180 than those on Day90 (**Fig 3**, dark blue dots), a fact that was attributed to an infection with SARS-CoV-2 on the period between 3–6 months after vaccination, and which was indeed verified with PCR. Additionally, IgG anti-S levels in the sera of four different donors (4/128 HCWs, 3%) were found higher from the previous sample, on Day270 (Fig 3, dark blue dots), indicating again an infection with the virus in the period between 6–9 months post-vaccination, which was verified with PCR for only two of them (2/4); the other two were asymptomatic. These post-vaccination infected individuals (8/131) were excluded from the naïve group titer analysis from those time points onwards. On Day180, despite the marked drop compared to Day42, anti-S IgG levels of the naïve HCWs were significantly higher than those on Day21, i.e., after the first dose (p<0.001), while on Day270 their levels did not differ significantly from Day21. On Day270 seropositivity for IgG anti-S Abs was around 93%, while Ab levels at that point, remain significantly higher from Day0 (p<0.001).

For anti-RBD IgG, mean levels on Day0 were 48±30, on Day21 319±270 and on Day42 1957±584 AU/well marking a significant increase in Ab levels (p<0.001) (**Fig 3**). The first dose of the vaccine led to 60.3% (79/131 naïve HCWs) anti-RBD seropositivity (p<0.001), while the second dose resulted in 100% seroconversion rate. On Day90 anti-RBD IgG mean levels were 1225±586 AU/well, showing a decline of 39.9% from Day42 (p<0.001). On Day180 mean levels dropped to 426±307 AU/well and on Day270 to 216±175 AU/well, marking a fall of 81.9% and 91.2% from Day42, respectively (p<0.001). On Day180 anti-RBD IgG levels of the naïve HCWs were significantly higher than those on Day21 (p<0.001). However, on Day270 anti-RBD levels displayed a significant drop from those on Day21 (p<0.05), in contrast to the anti-S IgG as described above. Additionally, on Day270 seropositivity for IgG anti-RBD Abs was around 43%, whereas Ab levels at that point, remained significantly higher from Day0 (p<0.001). As expected, the post-vaccination infected individuals (8/131) exhibited similarly higher anti-RBD IgG levels (Fig 3, dark red dots), validating the fact of an infection with SARS-CoV-2.

### Anti-viral IgA kinetics in response to two-dose BNT162b2 vaccination

For serum IgA Abs, we measured anti-S and anti-RBD levels on Day0 and Day42 for all naive HCWs cohort samples (n = 131). Mean levels of IgA anti-S Abs in naïve individuals were ranging from 53±33 AU/well on Day0 up to 660±404 AU/well on Day42 with 99.7% seroconversion, marking a significant increase in Ab levels (p<0.001). Additionally, mean levels of IgA anti-RBD Abs in naive individuals were ranging from 32±37 AU/well on Day0 up to 453±441 AU/well on Day42 with 96.3% of individuals had a positive seroconversion in IgA serum anti-RBD Abs, reaching more than 14-fold higher levels from Day0 (p<0.001).

**Fig 4:**
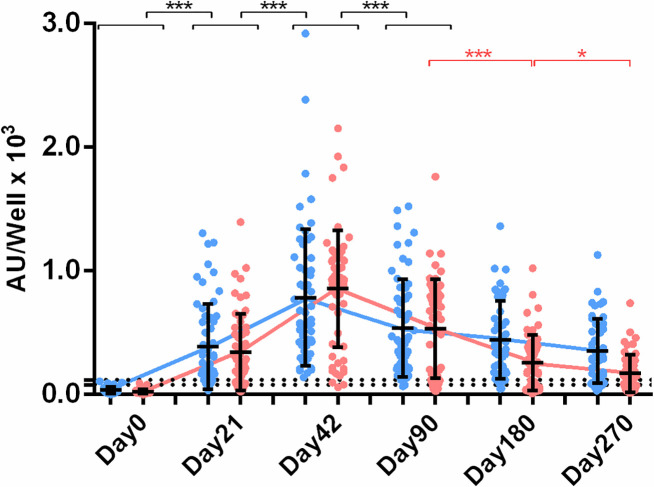
IgA anti-S and anti-RBD levels post-vaccination. Anti-S (blue) and anti-RBD (red) Abs were measured on Day0, 21, 42, 90, 180 and 270 (n = 56). Cut-off values for anti-S and anti-RBD Abs were 120 AU/well and 80 AU/well, respectively (dashed lines). Asterisks (***) indicate statistically significant differences (*p*-value < 0.001) between the compared groups. Each dot represents an individual, and error bars represent the mean value and standard deviation of the distribution.

Serum IgA anti-S and anti-RBD 9-month period kinetics were evaluated for 56 randomly selected individuals (male/female: 18/38) of our HCWs cohort (**Fig 4**). The peak of immune response was observed on Day42, and IgA Ab levels for both anti-S and anti-RBD Abs declined through time up to Day270. For both anti-S and anti-RBD Ab levels we found a significant decline (p<0.001) between every time point up to Day270. Similar to IgG Abs, IgA anti-S mean levels on Day270 were analogous to those on Day21, but still significantly higher than those on Day0. On the other hand, anti-RBD mean levels on Day270 were significantly lower (p<0.001) than those on Day21, although still significantly higher from Day0 (p<0.001). On Day270 IgA seropositivity for anti-S Abs was approximately 79%, while for anti-RBD Abs, about 53%.

### Follow-up analysis of anti-viral IgG and IgA Abs: Rate of Change (RC)

**Fig 5:**
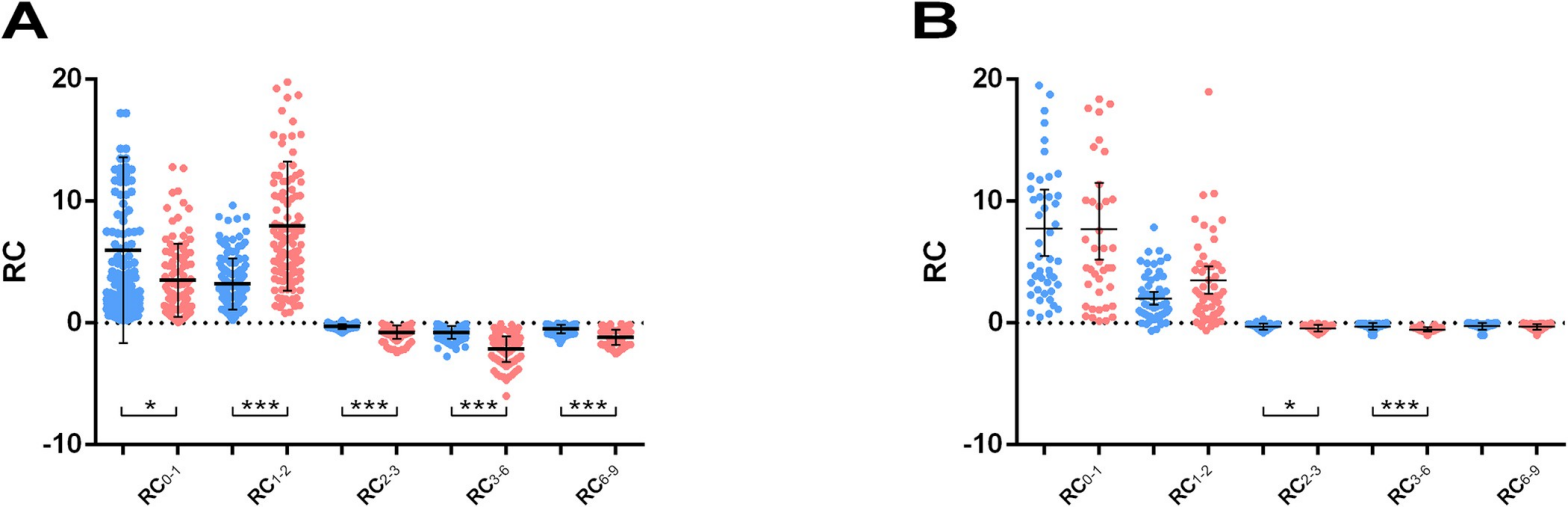
IgG and IgA rate of change up to 9-months post-vaccination. IgG (A) and IgA (B) RC values express the average change per day and were estimated for two subsequent time points: Day0-Day21 = RC_0–1_, Day21-Day42 = RC_1–2_, Day42-Day90 = RC_2–3_, Day90-Day180 = RC_3–6_, Day180-Day270 = RC_6–9_. Positive values indicate an increase in production, negative values imply a decrease, and values close to zero show no change in anti-S (blue) and anti-RBD (red) antibody binding. Asterisks (*) indicate statistically significant differences (*p*-value: *<0.05, ***<0.001). Each dot represents an individual, and error bars represent the mean value and standard deviation of the distribution.

Based on IgG and IgA anti-S and anti-RBD values, we calculated the RC index for further evaluation of the kinetics of these Ab responses. Since the time scale in this analysis was “days”, the units of the RC are SARS-CoV-2 binding Ab levels per day. Positive values refer to Ab production, negative values to Ab elimination, while values close to zero indicate no change in binding Ab values. **Fig 5** depicts the RC for IgG (A) and IgA (B), anti-S and anti-RBD binding Ab levels between sequential time-point pairs in the whole 9-month period. It is clear that the peak of anti-S Ab production was achieved between Day0 and Day21 (RC_0-1_), while the peak for Ab production to RBD was from Day21 to Day42 (RC_1-2_). The RC_0-1_ for anti-S Abs was significantly higher (p<0.05) from anti-RBD Abs, supporting that anti-S production was higher at that period. On the other hand, the RC_1-2_ between anti-S and anti-RBD exhibited statistically higher values for anti-RBD (p<0.001) indicating that anti-RBD Ab production took place mostly after the second dose of the vaccine. After Day42, RC of both Ab populations was in negative territory, reflecting the significant decrease in Ab levels. RC between IgG anti-S and anti-RBD Abs was significantly different (p<0.001) in all periods up to 9 months but for IgA Abs, significant differences were only observed between Day42 and Day90 (RC_2-3_, p<0.05) and between Day90 and Day180 (RC_3-6_, p<0.001). These data indicate a difference in anti-S and anti-RBD kinetics, which is more obvious for IgG than for IgA Abs.

### Factors affecting antibody response over time

The influence of several factors on anti-S and anti-RBD responses over time, namely previous COVID-19 infection, age, sex, medical history, BMI, previous flu or pneumococcal vaccination and occurrence of an adverse event upon vaccination, was also studied.

**Fig 6:**
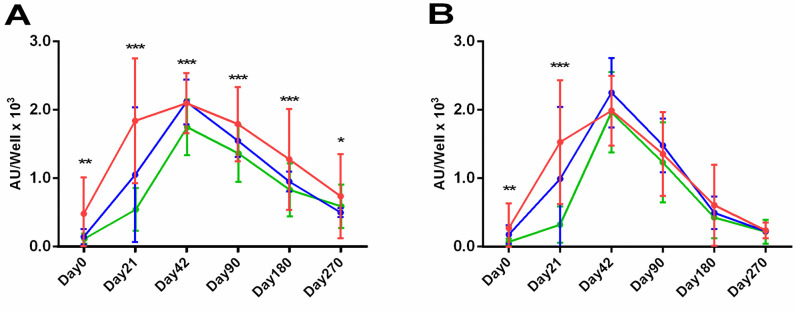
IgG anti-S and anti-RBD kinetics in a 9-month period: PCR+ individuals (red, n = 12 COVID-19), naïve individuals (green, n = 131) and suspected COVID-19 asymptomatic individuals (blue, n = 3). IgG anti-S (A) and anti-RBD (B) Abs were measured on Day0, 21, 42, 90, 180 and 270 days after the first dose. For each group, mean and standard deviation values are given for every time-point. Asterisks (*) indicate statistically significant differences (*p*-value: *<0.05, **<0.001, ***0.001) between the groups of PCR+ and naïve individuals.

Regarding previous SARS-CoV-2 infection, anti-S and anti-RBD Ab levels measured on every time point were higher in the PCR+ individuals (n = 12) and the suspected COVID-19 asymptomatic individuals (n = 3), compared to naïve vaccinated individuals (n = 131). For IgG Abs on Day0, mean value levels of PCR+ individuals were 473±531 and 275±362 AU/well for anti-S and anti-RBD, respectively (**Fig 6**). On Day42, mean values for anti-S and anti-RBD Abs reached 1832±911 and 1532±905 AU/well, respectively. Regarding the anti-S IgG Abs, on Day90 the mean levels for the PCR+ subjects were exactly equal with those of naïve individuals on Day42, while this effect remain evident up to Day270 with the Ab levels still being significantly higher for the PCR+ subjects (p<0.05) at every time point. This points out the strong influence of the previous SARS-CoV-2 infection on the production of anti-S Abs and their longer maintenance in the circulation.

Multivariate regression analysis results about the influence of several others factors (i.e. age, sex, medical history, BMI, previous flu or pneumococcal vaccination and occurrence of an adverse event upon vaccination) on anti-S and anti-RBD responses are cumulatively presented in **S2 Table**. Age and adverse events after vaccination are linked to anti-viral IgG and IgA Ab levels at various time points.

The age of the vaccinees appears to affect the response, with younger individuals presenting with higher levels of anti-viral IgG Abs. For example, on Day21 young individuals (<40 years old) had higher anti-S and anti-RBD IgG levels and on Day42 higher anti-S IgG and anti-RBD IgA levels compared to older age groups as confirmed by multivariate analysis (p<0.05). In addition, anti-S and anti-RBD IgG levels were higher at multiple time points for those participants who experienced adverse events following vaccination. Furthermore, previous vaccination for flu or pneumococcus appeared to be linked with higher anti-S IgG Ab levels on Day90 and Day180. Finally, the presence of chronic diseases and BMI>35 were found to negatively affect Ab levels, resulting in lower anti-S IgG, while male donors exhibited higher anti-RBD IgA levels compared to female donors (p = 0.047). However, the observations concerning chronic diseases, BMI and sex were only seen in a single time point, and thus, their significance needs to be further evaluated in future studies.

In addition to the above, we calculated and analyzed the Ab RC index from Day42 up to Day270. We found that anti-RBD IgG levels had a faster drop rate when compared to anti-S IgG (slope comparison: p<0.05), however this was not confirmed for IgA (slope comparison: p = 0.701). Furthermore, when multiple linear regression was assessed, using as dependent variables the factors of the analysis applied, adverse events after vaccination were found to be an important factor in maintaining anti-S IgG levels (p = 0.0335) (**S2 Fig**).

### IgG and IgA anti-viral responses after homologous (third) booster dose

**Fig 7:**
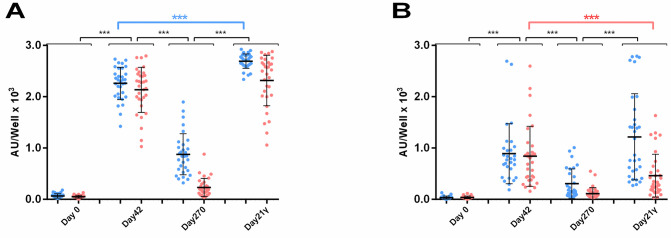
Antibody levels for 34 HCWs who received a third dose of the BNT162b2 mRNA vaccine. IgG (A) and IgA (B) anti-S (blue) and anti-RBD (red) Abs were measured on Day0, 42, 90, 270 days after the first dose on Day0 and on Day21γ, 21 days after the third dose. Asterisks (***) indicate statistically significant differences (*p*-value: <0.001). Each dot represents an individual and error bars represent the mean value and standard deviation of the distribution.

The effect of a third booster dose of BNT162b2 vaccine on Ab levels was evaluated for 34 HCWs. Blood sample was collected 21 days after the third dose (Day21γ) and tested in parallel with samples of Day0, Day42 and Day270 in order to comparatively evaluate the IgG and IgA anti-S and anti-RBD Ab levels (**Fig 7**). In all cases, we found a significant increase in Ab levels between Day270 and Day21γ. Interestingly, IgG anti-S Ab levels were significantly higher on Day21γ (2694±141 AU/well) compared to Day42 (2261±316 AU/well) (i.e. 21 days after the second dose), while IgG anti-RBD levels on Day21γ (2318±493 AU/well) had no significant difference compared to Day42 (2136±440 AU/well). Moreover, IgA anti-S Ab levels on Day21γ (1.008±676 AU/well) were at the same level with those on Day42 (838±555 AU/well) but anti-RBD Abs on Day21γ (475±426 AU/well) were significantly lower than those on Day42 (840±596 AU/well). These results indicate a more efficient production of anti-S than of anti-RBD Abs upon a third dose of BNT162b2 mRNA vaccine.

## Discussion

We present herein a planned 9-month follow-up study of serological response to mRNA homologous vaccination against COVID-19. We report the persistence of circulating anti-viral IgG and IgA Abs in a Greek HCW cohort prioritized for vaccination due to high risk of viral exposure, which coincided with the rapid deployment of the Pfizer/BioNTech BNT162b2 SARSCoV-2 vaccination in Greece [17]. Studies on humoral responses as a measure of biological protection conferred by the BNT162b2 mRNA vaccine, focus on IgG and less on IgA Ab dynamics [10,11], measured by commercial assays based on a single target antigen [8,10,11], whereas the even less studied IgM Abs are not indicators of long-term vaccine-acquired immunity against SARS-CoV-2 [20]. In contrast, the establishment of our in-house ELISAs offered the opportunity to simultaneously explore: a) the dynamics of IgG and IgA Abs, b) their binding capacity against the same viral epitopes (i.e., trimeric Spike protein and RBD), c) the humoral response in long-term time intervals, and d) the effect of homologous booster doses.

The diagnostic performance of anti-SARS-CoV-2 Abs to distinct epitopes may vary due to temporal kinetics. In serological surveys multiple Abs targeting different epitopes increase assay specificity and sensitivity [21–23]. Commercially available ELISAs commonly use the S1 fragment (~700 amino acids) as target antigen. Hence we decided to use a combination of trimeric S (1208aa), and its RBD portion (188aa), a major target of neutralizing Abs correlated with viral protection [2]. Those viral antigens have been previously utilized in ELISA and other immunoassays [5,18,19,23]. Rosado *et al*. have shown that although the anti-trimeric S IgG was the best-performing from all candidate tested biomarkers, the additional measurement of anti-RBD IgG increased the sensitivity of their method [23].

Following a single vaccine dose in immunologically naive recipients, anti-S and anti-RBD IgG Abs were detectable on Day21 in over 90% and 60% of individuals, respectively, while on Day42, 100% of individuals exhibited seropositivity along with maximum Ab levels for both antigens. Seropositivity persisted beyond six months’ post-vaccination, although the Ab levels were gradually decreasing. Dan M.J. *et al*. showed similar anti-S kinetics but sustained anti-RBD IgG Ab levels in a cohort of SARS-CoV-2 infected individuals [24]. Our results agree perfectly with multiple studies worldwide, suggesting that racial differences do not represent a variable affecting significantly humoral response to mRNA vaccines [25]. Matusali G. *et al*. reported persistent levels of anti-trimeric S IgG and anti-RBD IgG up to 6 months after the second COVID-19 vaccine dose with the anti-RBD IgG Abs presenting a steeper decay [26]. Terpos *et al*. described also in a Greek cohort that 9 months after the second dose the anti-RBD levels were around 25% of those observed at 2 weeks after full vaccination, even though neutralizing Abs were still detectable in high percentages for the majority of the vaccinees, using GenScript’s cPass^TM^ SARS-CoV-2 NAbs Detection Kit [27]. In contrast, in our case on Day270, an ever-greater decline of almost 90% of the anti-RBD levels was observed, and only 30% of the vaccinees exceeded the threshold of seropositivity for anti-RBD Abs, which represent the main source of neutralizing Abs. However, anti-S response persisted above the threshold of positivity for about 80% of the vaccinees. The faster drop rate of anti-RBD compared to anti-S Ab levels could similarly be explained by a narrower anti-RBD Ab repertoire, targeting a restricted number of epitopes compared to those on trimeric Spike ectodomain. While the RC index has been shown to be significantly higher in cohorts of vaccinees receiving either mRNA or adenovirus vector-based COVID-19 vaccines compared to infected individuals, several hypotheses have been made trying to explain this fastest decline, such as the lack in number and diversity of memory B-cells and of bone marrow plasma cells [28,29]. According to our data, although the first vaccine dose resulted in a positive RC index, specifically for anti-S Abs, a significant increase in the RC index for anti-RBD Abs was especially observed between Day21 and Day42, reflecting the importance of the second vaccine dose for the efficient production of anti-RBD Abs. Our evidence implies ongoing immune protection against COVID-19, as also described elsewhere, but still points out the need for a booster dose, as during the pandemic high Ab levels of wide repertoire are needed for the members of the community, in order to achieve efficient protection from severe COVID-19 [24,27].

In addition to IgG, our data demonstrate that BNT162b2 mRNA vaccine also elicits antigen-specific IgA, which may be important in preventing transmission as well as infection [30,31]. There is a strong evidence that IgA has a protective effect against SARS-CoV-2 infection, while isolated secretory IgA has been proven to neutralize efficiently the virus [12]. Although the general notion is that intramuscular vaccines do not elicit effective IgA immune responses at the mucosal surfaces, studies have described the presence and long-term persistence of IgA anti-SARS-CoV-2 Abs in the nasal mucosa and the breast milk of vaccine recipients [15,32]. IgA specific Abs have been detected even in the cerebrospinal fluid of vaccinees and infected individuals, after admission in the hospital due to neurological symptoms [33], although longitudinal studies are lacking. Serum IgA Ab levels represents a useful biomarker reflecting the development of humoral immunity correlated with neutralizing activity in secretions at the mucosal surface [31]. Indeed, as also reviewed by Sheikh-Mohamed *et al*.”, mRNA vaccination (e.g., BNT162b2) leads to the development of robust RBD-specific Abs with neutralizing ability in the saliva, while the specific IgG and IgA levels exhibit similar dynamics to those, we observed in the sera of our cohort [34]. However, delayed Ab responses are more evident for the IgA anti-SARS-CoV-2 Abs generated by vaccination when compared to infection alone [20,35,36], supporting the need of the second vaccine dose in order to achieve efficient mucosal protection [15,37,38]. In our study, anti-viral IgA Ab kinetics similarly to IgG, reached maximum levels on Day42 but dropped thereafter quickly to lower levels of seropositivity up to 9 months. Our data are in accordance with studies that used commercially available ELISA kits and showed a significant decrease in IgA levels 3 months after vaccination for most participants, while afterwards their levels approached zero levels [14]. However, we observed that SARS-CoV-2 IgA Ab responses remained above the positivity threshold levels (79% for anti-S and 53% for anti-RBD) even on Day270.

Along with time, differences regarding the kinetics of immune responses can be also caused by homologous *vs*. heterologous vaccinations, a major matter of concern in many updated studies. Data from other studies have confirmed that heterologous vaccination triggers a stronger Ab production than two doses of a single vaccine [39]. Additionally, infection or a combination of infection and vaccination induces more robust and durable antigen-specific IgG and IgA Abs [40]. As high levels of anti-viral Abs have been previously correlated with enhanced inhibitory capacity against variants of concern, our data alongside with other studies, highlight the importance of the third dose, since anti-viral Abs were significantly elevated and reached analogous or even greater levels of those after the second vaccine dose. Homologous and heterologous vaccine regimens displayed no significant differences regarding the humoral or cellular immune response after the third or subsequent booster vaccine doses [41]. As a homologous third dose was also included in this study, we observed an even bigger increase in production of IgG anti-S and analogous levels of IgA anti-S Abs compared to Day42. The anti-RBD IgG expression was also enhanced but the effect didn’t exceed the production dynamics of the second dose, while anti-RBD IgA levels were significantly lower than those (Day42), suggesting a weaker IgA mediated Ab enhancement of booster doses along with reduced neutralization capacity at mucosal surfaces [15].

One more parameter that significantly affects post-vaccination anti-viral IgG and IgA kinetics is the type of the vaccine, as different candidates are globally available. Studies on different vaccine types report that mRNA vaccines induced the highest amount of anti-S IgG and IgA Abs and a high serum neutralization potential when compared to vector based vaccines (e.g., Astra Zeneca, Janssen, Sputnik-V) or vaccines using whole inactivated SARS-CoV-2 viral particles (Sinopharm) [29]. All types of vaccination also induced spike-specific IgA secretion, indicating individual mucosal immunity against SARS-CoV-2 [29]. We observed highly increased IgA levels for some individuals already after the first vaccination with BNT162b2, arguing for a highly individual variance in class switch towards IgA. On the other hand, the vector-primed vaccination induces a more potent cellular response [42]. Among vector-based vaccines, a higher IgA expression was seen in individuals vaccinated with Sputnik-V, while Janssen vaccine exhibited a rather poor performance. On the other hand, Sinopharm vaccine had an even weaker performance in terms of neutralizing Ab induction [29]. These observations for adenovirus-vectored and virus-inactive vaccines, particularly in older vaccinees, may partially be explained by cross-reactive Abs generated by previous adenoviruses natural infections or vaccinations [43].

Although the aim of our study was to analyze naïve vaccinees, the few pre-infected individuals of this HCWs cohort, were also analyzed in parallel as we considered their report necessary. We verified as expected that previous infection further enhances the strength of Ab responses in vaccine recipients, a fact that can be attributed to the adaptive memory immune reactions [37,38]. In accordance with our findings, Glück *et al*. reported in a HCWs cohort, declining but still detectable anti-RBD IgG and IgA Abs 6 months after the second dose, while boosted COVID-19 convalescents exhibited enhanced and more stable Ab levels in the time course [38]. Moreover, Desmecht *et al*. reported enhanced anti-S IgG Ab responses, which endure even one year after vaccination when combined with previous infection, comparing with natural infection or vaccination alone [37]. Overall, the accumulating evidence of our study and others’ shows more intense long-term humoral response and significantly higher vaccine effectiveness in previously infected persons from that of naive recipients of two vaccine doses [20,37,38].

Surprisingly, we observed a positive association between previous flu vaccination and anti-SARS Ab responses that remain eminent up to Day270. This observation leads to the assumption that a heterologous immune challenge could lead to enhanced responses but this hypothesis should be further investigated. In addition, previous infection with pneumococcal or other pathogens, as well as the presence of commensal microbiota *per se*, have been described to confer heterologous immune protection via the production of cross-reactive T-cells and Abs shaping a possible mechanism explaining anti-SARS immune protection in subjects unexposed to the virus [44–46]. Interestingly, Tan *et al*. showed that previously infected individuals with SARS-CoV-1, exhibited enhanced humoral response after vaccination with mRNA vaccines (e.g., BNT162b2) and possess potential neutralizing activity against other corona viruses [47]. The effect of SARS-CoV-2 vaccination on MERS-CoV survivors to our knowledge has barely been studied [46]. On the other hand, our findings on the effect of previous flu vaccination are supported by other groups, who also found enhanced humoral response, neutralizing Abs and cellular immunity but still without analyzing long-term specific Ab dynamics [44,46]. Additionally, our study provides data on the long-term kinetics of the specific IgG and IgA, anti-S and anti-RBD Abs positively correlated with previous Influenza vaccination indicating pre-existing cross-reactive B-cells as well as the potential existence of naturally occurring or elicited polyreactive Abs implicated in viral protection [46,48].

Age, BMI, and the presence of chronic diseases were major factors affecting the humoral responses to BNT162b2 mRNA vaccine over time, as it has also been shown in the literature [43]. This is in accordance with the previously reported finding that a more robust immune response is associated with the presence of an adverse event upon vaccination [43]. In our cohort, regarding the sex ratio, superior number of female subjects is in agreement with equivalent percentages (about 70%) of females among HCWs worldwide, as reported by WHO [49]. Taking this limitation into account, statistical analysis didn’t reveal any major difference among IgG Abs, but our data showed a superior production of anti-S and anti-RBD IgA Abs in males on Day42. In contrast, other studies support superior anti-viral IgΑ Abs in females instead, which is probably attributed to different cohort distributions [14].

A low percentage of fully vaccinated BNT162b2 recipients still develop symptomatic SARS-CoV-2 infection [34]. In our study, all the post-vaccination infected individuals (n = 8, 6%) had mild or no COVID-19 symptoms and belonged to all age groups of the study. These breakthrough infections were attributed to high viral loads exposure of these individuals from hospitalized patients. Nevertheless, they all had mild symptoms. Notably, they all displayed anti-S and anti-RBD Ab levels well above the seropositivity threshold before infection. Overall, our findings further support that BNT162b2 is an effective vaccine protecting from severe disease phenotype.

In conclusion, our combinatorial protocol with the use of the trimeric S protein along with its RBD targeted by both, IgG and IgA specific Abs provides a more accurate overview on anti-SARS-CoV-2 Ab repertoire and kinetics. Besides BNT162b2 effectiveness, anti-viral Abs decline over time, and these data along with other studies support the necessity of booster doses in order to sustain an efficient anti-viral protection. [14,27]. Moreover, as the pandemic phase weakens and eventually COVID-19 will enter an epidemic phase, our data indicate that as seropositivity following BNT162b2 vaccination remains well above threshold up to 9 months, the gap period between booster doses could be set in longer time intervals, perhaps annually, relieving individuals and countries from the fear and anxiety created by the pandemic. Moreover, heterologous vaccination and/or updated vaccines give rise to greater neutralizing potentials primarily towards emerging variants of concern and therefore should considered as an effective alternative. Follow-up studies of anti-viral Ab levels after receiving a third and subsequent booster doses, will highlight the degree of protection over time, as it is considered critical for the pandemic management and post-pandemic era.

## Supporting information

S1 Fig ELISA against N protein for pre-pandemic, naive and PCR+ individuals. (TIF)

S2 Fig Slope comparison for anti-S and anti-RBD IgG antibodies from Day42 up to Day270. (TIF)

S1 Table RAW data. (XLS)

S2 Table Multivariate regression analysis results for other factors affecting the immune response to vaccination. (PDF)
