# Supplementary figures and images for: Combined monitoring of IgG and IgA anti-Spike and anti-Receptor binding domain long term responses following BNT162b2 mRNA vaccination in Greek healthcare workers
Source: PLoS One. 2022 Nov 21;17(11):e0277827. doi: 10.1371/journal.pone.0277827 (PMC9678302; doi:10.1371/journal.pone.0277827)

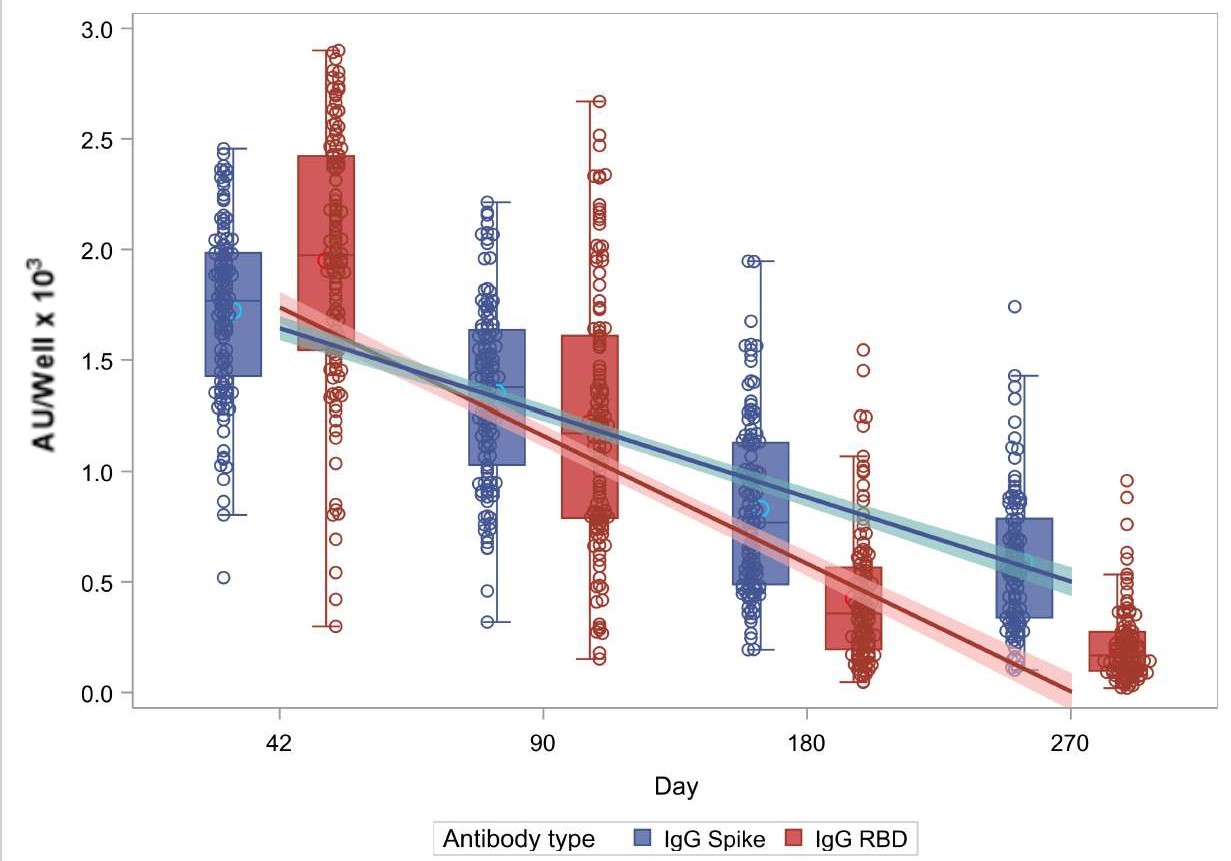

Supplement: S2 Fig — (TIF) [file pone.0277827.s002.tif]
