# Supplementary material for: Combined monitoring of IgG and IgA anti-Spike and anti-Receptor binding domain long term responses following BNT162b2 mRNA vaccination in Greek healthcare workers
Source: PLoS One. 2022 Nov 21;17(11):e0277827. doi: 10.1371/journal.pone.0277827 (PMC9678302; doi:10.1371/journal.pone.0277827)
Supplement: S2 Table — (PDF) [file pone.0277827.s004.pdf]

		Day 21			Day 42			Day 90			Day 180			Day 270		
	Parameter	Effect size	SE	p	Effect size	SE	p	Effect size	SE	p	Effect size	SE	p	Effect size	SE	p
IgG Spike	Gender (female vs. male)	0.0241	0.0614	0.6957	-0.0184	0.0775	0.8133	0.0224	0.0808	0.7818	0.0221	0.0803	0.7834	-0.0548	0.0729	0.4543
	BMI≥35 (yes vs. no)	-0.1573	0.1030	0.1291	-0.2218	0.1295	0.0895	-0.2700	0.1350	**0.0477**	-0.1494	0.1299	0.2525	-0.0457	0.1194	0.7028
	Chronic disease (yes vs. no)	-0.1246	0.0610	**0.0433**	-0.0531	0.0759	0.4854	-0.0377	0.0791	0.6345	-0.0868	0.0773	0.2640	-0.0653	0.0678	0.3382
	Adverse event (any) (yes vs. no)	0.0133	0.0569	0.8157	0.0771	0.0839	0.3604	0.1567	0.0875	0.0757	0.1773	0.0843	**0.0377**	0.2064	0.0795	**0.0110**
	Vaccination (flu or pneumococcus) (yes vs. no)	0.0560	0.0558	0.3170	0.1341	0.0721	0.0651	0.2355	0.0751	**0.0022**	0.1922	0.0733	**0.0100**	0.0979	0.0653	0.1370
	Age	-0.0046	0.0025	0.0747	-0.0102	0.0032	**0.0017**	-0.0066	0.0033	**0.0476**	-0.0032	0.0035	0.3683	-0.0063	0.0031	**0.0403**
IgG RBD	Gender (female vs. male)	0.0309	0.0534	0.5634	-0.1109	0.1118	0.3234	-0.1254	0.1135	0.2714	-0.0547	0.0626	0.3838	-0.0637	0.0398	0.1132
	BMI≥35 (yes vs. no)	-0.0946	0.0895	0.2929	-0.3459	0.1868	0.0666	-0.2317	0.1896	0.2240	-0.0588	0.1011	0.5619	-0.0225	0.0652	0.7302
	Chronic disease (yes vs. no)	0.0011	0.0531	0.9833	-0.0145	0.1094	0.8946	-0.0288	0.1110	0.7961	-0.1124	0.0602	0.0646	-0.0593	0.0370	0.1126
	Adverse event (any) (yes vs. no)	0.0523	0.0495	0.2926	0.2801	0.1210	**0.0224**	0.3600	0.1228	**0.0040**	0.2040	0.0657	**0.0024**	0.1344	0.0434	**0.0026**
	Vaccination (flu or pneumococcus) (yes vs. no)	0.0637	0.0485	0.1912	0.1352	0.1039	0.1956	0.1683	0.1054	0.1130	0.0622	0.0571	0.2787	0.0185	0.0356	0.6055
	Age	-0.0050	0.0022	**0.0267**	-0.0089	0.0046	0.0551	-0.0038	0.0047	0.4126	0.0001	0.0027	0.9611	-0.0018	0.0017	0.2721
IgA Spike	Gender (female vs. male)	0.0126	0.1112	0.9102	-0.1606	0.0851	0.0616	0.0076	0.1136	0.9467	0.0864	0.0970	0.3781	0.1486	0.1037	0.1594
	BMI≥35 (yes vs. no)	-0.0758	0.1582	0.6340	0.0342	0.1354	0.8009	-0.2004	0.1586	0.2126	-0.1701	0.1348	0.2134	-0.0301	0.1483	0.8400
	Chronic disease (yes vs. no)	0.0879	0.1042	0.4033	0.0285	0.0816	0.7274	0.0841	0.1039	0.4222	0.0049	0.0901	0.9567	0.0104	0.0952	0.9136
	Adverse event (any) (yes vs. no)	-0.1781	0.1102	0.1127	0.0375	0.0971	0.7003	-0.1825	0.1265	0.1558	-0.0006	0.1076	0.9958	-0.0196	0.1100	0.8592
	Vaccination (flu or pneumococcus) (yes vs. no)	0.0599	0.1080	0.5817	0.0389	0.0789	0.6231	0.0101	0.1086	0.9261	-0.0275	0.0929	0.7688	-0.0601	0.0976	0.5411
	Age	-0.0031	0.0047	0.5135	0.0019	0.0035	0.5842	0.0085	0.0047	0.0748	0.0059	0.0042	0.1662	0.0052	0.0042	0.2265
IgA RBD	Gender (female vs. male)	0.1666	0.0885	0.0660	-0.1837	0.0914	**0.0469**	0.0827	0.1252	0.5122	0.0809	0.0706	0.2576	0.0582	0.0444	0.1966
	BMI≥35 (yes vs. no)	-0.2094	0.1259	0.1031	-0.0799	0.1454	0.5840	-0.0067	0.1747	0.9697	-0.0317	0.0980	0.7481	0.0183	0.0635	0.7740
	Chronic disease (yes vs. no)	-0.0568	0.0830	0.4971	0.0767	0.0876	0.3835	-0.0534	0.1144	0.6431	-0.0917	0.0655	0.1684	-0.0397	0.0408	0.3362
	Adverse event (any) (yes vs. no)	-0.0339	0.0877	0.7007	0.0057	0.1043	0.9568	-0.1477	0.1394	0.2947	0.0530	0.0783	0.5020	0.0095	0.0471	0.8403
	Vaccination (flu or pneumococcus) (yes vs. no)	0.0901	0.0860	0.3000	0.1264	0.0848	0.1387	0.0641	0.1197	0.5944	-0.0120	0.0676	0.8600	0.0228	0.0418	0.5877
	Age	-0.0029	0.0038	0.4462	-0.0060	0.0038	0.1110	0.0041	0.0051	0.4316	0.0013	0.0030	0.6792	-0.0017	0.0018	0.3618
